# Histological Value of Duodenal Biopsies

**DOI:** 10.1100/tsw.2005.44

**Published:** 2005-05-10

**Authors:** Limci Gupta, Hamid B.

**Affiliations:** Countess of Chester Hospital NHS Foundation Trust, Chester, Cheshire, CH2 1UL, UK

**Keywords:** duodenal biopsies, history on the form, coeliac disease, serology, histopathological diagnosis of value

## Abstract

This study was performed to see the value of histopathological diagnosis in management of patients with duodenal biopsies; to look for correlation of histology and serology in suspected cases of coeliac disease; the reasons for taking duodenal biopsies and whether proper adequate histories are provided on the forms sent with request for histopathological view on duodenal biopsies. Here are the observations of the study followed by the discussion.

## INTRODUCTION

Coeliac disease is a permanent gluten-sensitive enteropathy and is associated with various autoimmune disorders[1]. It is also a premalignant condition with an association with abdominal lymphoma and small bowel adenocarcinoma. This is a condition that is diagnosed definitively by histology, although various serological tests with 75–95% sensitivity are available[2].

We performed a study of 100 cases of duodenal biopsies at our department:

1. To study the inadequacy rates of the histories provided on the clinical forms sent with the duodenal biopsies.
2. To look for the reasons for taking the duodenal biopsies.
3. To study the correlation of serology with histology in suspected cases of coeliac disease.
4. To study whether the histology of duodenal biopsies is of diagnostic importance in routine practice and especially in coeliac disease.

## MATERIALS AND METHODS

Data of 100 consecutive cases of duodenal biopsies were collected on Meditech and Medisec Net in the Department of Histopathology, Countess of Chester Hospital. The cases were from November 2004 until January 2005.

## OBSERVATIONS AND RESULTS

1. Most common symptoms were anaemia, diarrhoea, abdominal pain, malabsorption, or weight loss (Graph 1).
2. Only 36 patients out of the above 100 cases had serology for coeliac.
3. Out of these 36 patients, 11 were positive for serology of which only 3 had positive histology for coeliac disease (27% correlation with positive serology) (Graph 2).
4. Two patients had low total IGA levels out of six total coeliac cases (two were for surveillance, which did not show coeliac changes on surveillance biopsies) giving a 33% correlation.

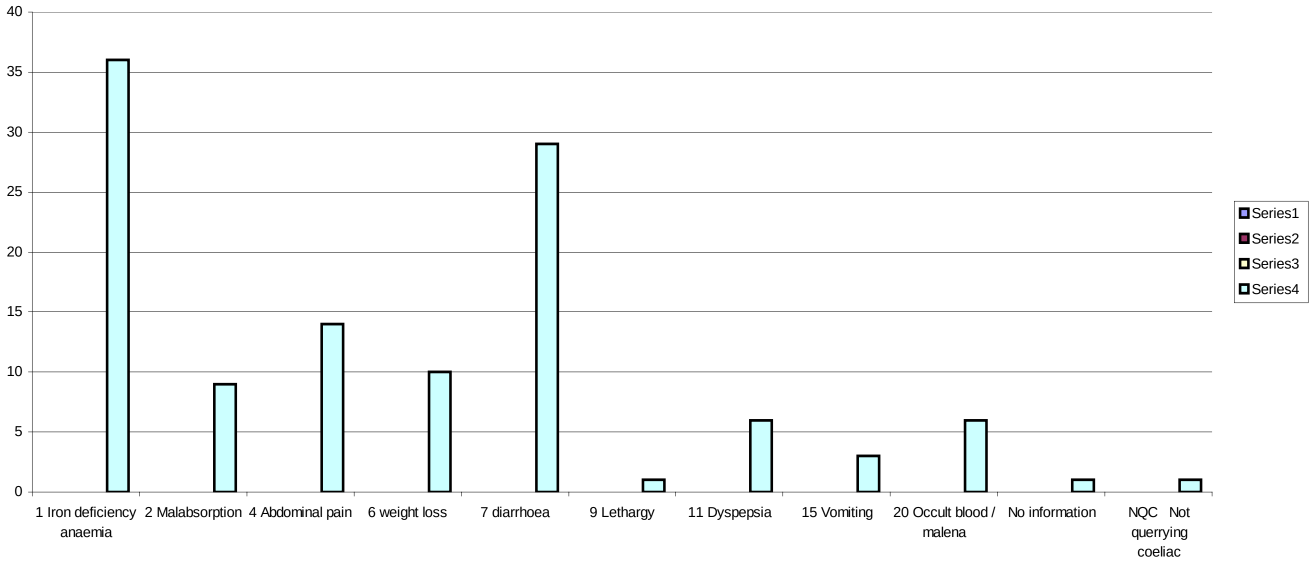

**Graph 1**

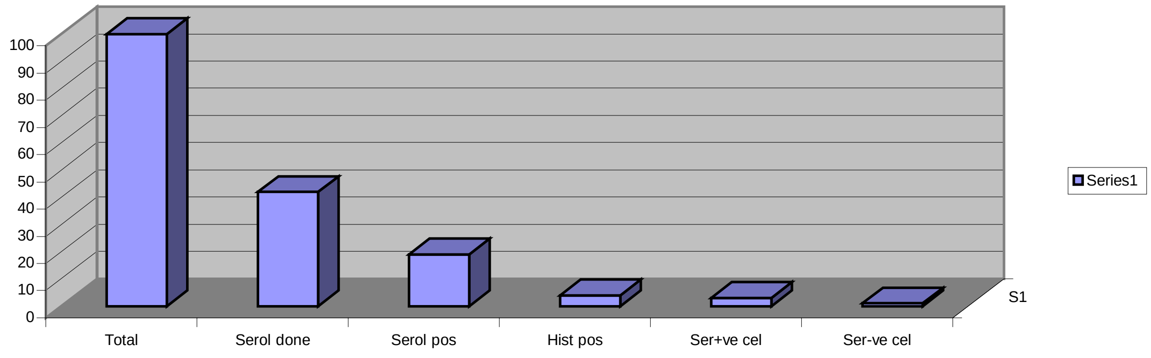

**Graph 2**

5. The age range for duodenal biopsies in this study was 16–84 years. Maximum numbers were between 41 and 70 years of age (Graph 3).
6. One positive coeliac (out of four) on histology was negative for serology giving a correlation of 75%.
7. Out of 82 cases, 4 had positive histology for coeliac disease giving a sensitivity of 5%.
8. Of the cases, 14 had unjustified reasons for taking duodenal biopsies (with no specific symptoms for coeliac disease and with no serology asked for coeliac).
9. There were 64 cases that were provided with adequate history, but 36 could have been provided with more useful information.

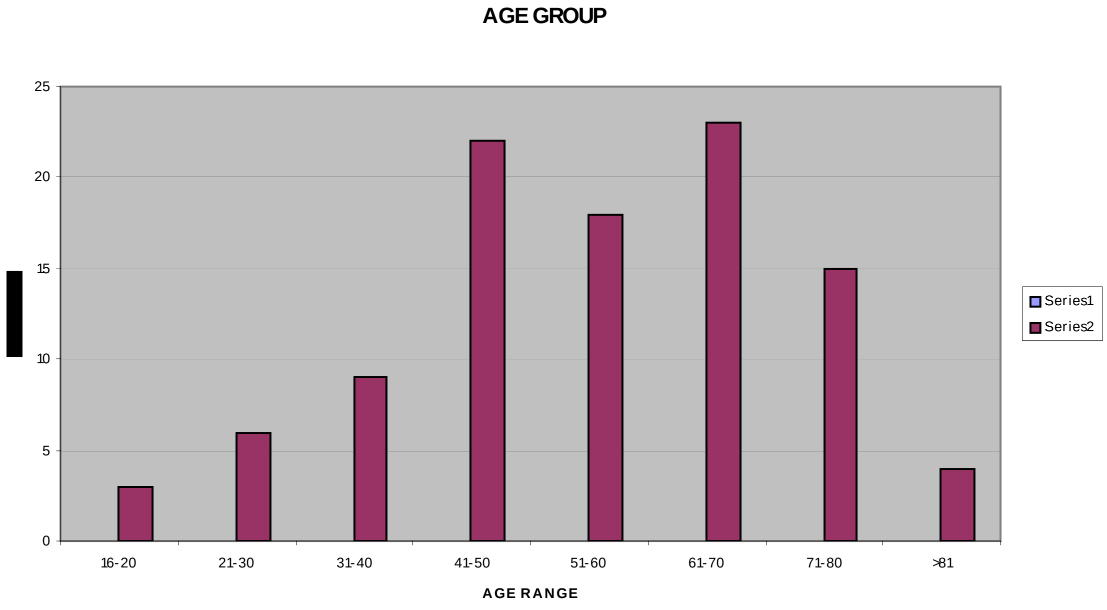

**Graph 3**

10. Histology reports: coeliac disease, 4; mild duodenitis, 2; normal, 92; insufficient for diagnosis, 1; other specific disease, 1. Two cases showed mild duodenitis (one with clinical suspicion of coeliac and one with other associated conditions). One showed ectopic gastric mucosa. This gives a 7% figure for diagnosing any kind of abnormality on histology of duodenal biopsies.
11. No MedisecNet notes were available in 9 out of these 100 cases.
12. Out of 91 cases, 18 were positive for diabetes of which only 1 out of 6 coeliac patients had diabetes.
13. Out of 91 cases, 6 had thyroid abnormality and none of the coeliacs had it.

## DISCUSSION

One out of four cases with positive serology showed normal duodenal histology giving a figure of 75% correlation, which is in accordance with the study by Gregor[3]. These results emphasize the usefulness of serology in the diagnosis of coeliac disease. Also, it states a 2.7% chance of negative serology with a positive histology. Three out of 18 cases showed other pathology out of which one showed ectopic gastric mucosa giving diagnostic specificity in other conditions as 17%. Therefore, with suspicion of conditions other than coeliac disease, in mild changes on endoscopy, duodenal biopsies for histology again are useful as diagnostic specificity is 17% in such cases.

If there is any suggestion of coeliac disease as a cause of anaemia, duodenal biopsies for confirmation must be done, keeping in mind the associations of this condition with malignancies and autoimmune disorders[3]. In our study, the age range for coeliac disease is above 40–80 years, indicating a shift towards older age groups, compared to past studies. Therefore, histological examination of duodenal biopsies is useful and should be continued as in the past.
